# STAT3 inhibitor Stattic Exhibits the Synergistic Effect with FGFRs Inhibitor Erdafitinib in FGFR1-positive Lung Squamous Cell Carcinoma

**DOI:** 10.7150/jca.97477

**Published:** 2024-08-19

**Authors:** Hongqin Zhong, Ling Wang, Xue Zhu, Shu Li, Xiyue Li, Chen Ding, Ke Wang, Xun Wang

**Affiliations:** 1 NHC Key Laboratory of Nuclear Medicine, Jiangsu Key Laboratory of Molecular Nuclear Medicine, Jiangsu Institute of Nuclear Medicine, Wuxi 214063, Jiangsu Province, China.; 2 Department of Radiopharmaceuticals, School of Pharmacy, Nanjing Medical University, Nanjing 211166, Jiangsu Province, China.; 3 Department of Pulmonary and Critical Care Medicine, Jiangnan University Medical Center, Jiangnan University (Wuxi No.2 People's Hospital) Wuxi 214126, Jiangsu Province, China.; 4 Nantong University Medical School, Nantong 226007, Jiangsu Province, China.; 5 Wuxi School of Medicine, Jiangnan University, Wuxi 214126, Jiangsu Province, China.

**Keywords:** Lung Squamous Cell Carcinoma, Erdafitinib, Stattic, FGFR1, STAT3

## Abstract

Lung squamous cell carcinoma (LUSC), a subset of non-small cell lung cancer (NSCLC), accounts for about 30% of all lung cancers (LC) and exhibits a dismal response to current therapeutic protocols. Existed studies have indicated that aberrations in fibroblast growth factor receptors (FGFRs) play a pivotal role in the progression of LUSC, rendering them as attractive targets for therapeutic intervention in this cancer type. This study found that Erdafitinib (Erda), a novel pan-FGF receptor tyrosine kinase inhibitor (TKI), exerted a cytotoxic effect on LUSC cells. However, STAT3, the downstream target of FGFRs, remained still activated despite Erdafitinib treatment. Then, a STAT3 inhibitor, Stattic (Sta), was concurrently used with Erdafitinib, and the combined treatment demonstrated a synergistic efficacy in both *in vitro* and *in vivo* models of LUSC when compared to that of the treatment of the Erdafitinib or Stattic alone. Further molecular studies showed that such an effect of Erdafitinib and Stattic was associated with their concurrently inhibitory effect on FGFR1 and STAT3 signaling in LUSC cells. Therefore, the findings of this study indicated that the concurrent use of Erdafitinib and Stattic is a promising therapeutic approach for the treatment of FGFR1-positive LUSC.

## Introduction

Lung cancer is the most diagnosed malignancy and a leading cause of cancer-associated mortality 1, 2. Non-small cell lung cancer (NSCLC) accounts for ~85% of lung cancer cases, ~30% of which are lung squamous cell carcinoma (LUSC) 3. The treatment of LUSC often involves a combination of therapies including surgery, chemotherapies, and radiation therapy. However, there remains a high mortality of LUSC, because there are few approved targeted therapies available for this cancer type 4. Thus, there is an urgent need to develop effective therapeutics for LUSC. Fibroblast growth factor receptors (FGFRs) are a family of tyrosine-kinase receptors. Their signaling cascade regulates cellular proliferation, differentiation, and survival 5, 6. Deregulation of the FGFR signaling has been recognized as a driving force for tumor progression 7. Comprehensive genomic studies have shown that aberrations in the FGFR signaling are present in various cancer types including LUSC, suggesting that FGFRs may be potential drug targets for the relevant cancer types 8, 9. Noteworthy, several selective FGFRs inhibitors are under investigation in clinical trials of LUSC 10, 11.

Erdafitinib is a novel pan-FGF receptor tyrosine kinase inhibitor (TKI) approved by the U.S. Food and Drug Administration (FDA), to treat FGF-positive locally advanced, unresectable or metastatic uroepithelial carcinoma 12, 13. Our previous study has reported that Erdafitinib acts as a CDK2 inhibitor to suppress the growth of human lung adenocarcinoma cells by inducing cell cycle S phase arrest 14. Giacomini *et al.* have also shown that Erdafitinib has anti-cancer effect in the FGF-dependent lung cancer cells through inhibiting the FGF/FGFR signaling 15. However, we found that Erdafitinib only had a moderate inhibitory effect on LUSC cells, possibly due to an ineffective inhibitory effect on STAT3, a downstream target of FGFRs. We then further investigated the combined therapy of Erdafitinib and a STAT3 inhibitor Stattic in LUSC cells, which demonstrated a synergistic effect in *in vitro* and *in vivo* LUSC models.

## Materials and methods

### Chemicals and reagents

Erdafitinib (purity > 99%) was obtained from Qianyan Biotech (Shanghai, China) and dissolved in dimethyl sulfoxide (DMSO). Chemicals such as Stattic were obtained from MCE (Shanghai, China). Reagents such as antibodies were used as follows: FGFR1 (1:500, ab76464, Abcam, MA, USA), p-FGFR1 (1:1000, ab173305, Abcam), FGFR2 (1:1000, ab109372, Abcam), p-FGFR2 (1:2000, abs140266, Absin, Shanghai, China), FGFR3 (1:2000, ab133644, Abcam), p-FGFR3 (1:200, abs140268, Absin), FGFR4 (1:1000, ab178396, Abcam), p-FGFR4 (1:2000, abs1039979, Absin), AKT (1:1000, ab185633, Abcam), p-AKT (1:1000, ab192623, Abcam), ERK1/2 (1:1000, AF1051, Beyotime, Nantong, China), p-ERK1/2 (1:1000, AF1891, Beyotime), STAT3 (1:1000, ab68153, Abcam), p-STAT3 (1:1000, ab267373, Abcam), E-Cadherin (1:1000, sc-8426, Santa Cruz Biotechnology, CA, USA), Vimentin (1:1000, sc-373717, Santa Cruz Biotechnology), GAPDH (1:2000, D190090, Sangon Biotech, Shanghai, China), HRP-labeled Goat Anti-Mouse IgG(H+L) (1:1000, A0216, Beyotime), HRP-labeled Goat Anti-Rabbit IgG(H+L) (1:1000, A0208, Beyotime). Other materials were obtained from Sangon (Shanghai, China) and Beyotime (Nantong, China).

### Cell culture and transfection

The human lung squamous carcinoma H520 cells and human lung epithelial BEAS-2B cells were obtained from the National Collection of Authenticated Cell Cultures (Shanghai, China). Cells were cultured in RPMI1640 with 10% (v/v) fetal bovine serum (FBS) and penicillin/streptomycin (P/S) in a humidified atmosphere of 5% CO_2_ at 37℃. For the co-treatment of Erdafitinib and Stattic, the two compounds were mixed at certain concentrations and then added to the cell culture (24 h treatment). For FGFR1 knockdown, cells were transfected with FGFR1 siRNA (sc-57132, Santa Cruz Biotechnology), using Lipofectamine 2000 (Life Technologies, CA, USA) according to the manufacturer's protocols 16. After 48 h, Western blot analysis was performed to determine the efficiency of transfection. In addition, cells underwent subsequent experiments 48 h post‑transfection.

### Cell viability assay

MTT (3-(4,5-dimethylthiazol-2-yl)-2,5-diphenyltetrazolium bromide) (ST1537, Beyotime) assay was conducted to determine cell viability. After treatment, cells were incubated with 5 mg/mL MTT (20µL) solution for 4 h at 37℃. Following that, 150 µL of DMSO was added to each well and the plates were mixed on an orbital shaker for 10 min at room temperature. The absorbance was measured at 490 nm using a SpectraMax M5 microplate reader (Molecular Devices, CA, USA).

### Synergism calculation

Cells were treated with varying concentrations of Erdafitinib and Stattic. Finally, the MTT assay was performed and the SynergyFinder package 20 was used to calculate the combination index (CI), which reflected the type of drug interaction 17. The CI value less than 1 indicated a synergistic effect of the two drugs.

### Colony formation assay

A colony formation assay was further conducted to determine cell viability. After treatment, cells were seeded into 6-well plates and cultured for 11-14 days in a humidified atmosphere of 5% CO_2_ at 37℃. Then, cells were fixed in methanol for 15 min, and cell colonies were stained with crystal violet for 15 min at room temperature. Upon air drying, the number of colonies was counted using an inverted phase contrast microscope (Olympus IX53, Tokyo, Japan).

### Cell apoptosis analysis

Cell apoptosis was conducted with Annexin V-FITC/PI double staining detection kit (abs50001, Absin). After treatment, cells were harvested and fixed in 70% ethanol on ice for 30 min, and digested with 100 µg/mL of ribonuclease A for 20 min at 37 ℃. Then, Annexin V-FITC and PI were added for staining for 20 min in the dark. The samples were analyzed with flow cytometry (Becton-Dickinson, CA, USA) immediately and data were analyzed using FlowJo software (Becton-Dickinson).

### Cell metastasis assays

Cell migration assay was conducted as follows: cells were spread in 6-well plates, which were pretreated with 0.1 mg/mL Poly-D-lysine hydrobromide at 37 ℃ for 1 h and grown until 80-90% confluence. Cells were evenly distributed and created an artificial wound of approximately 900 μm by a pipette tip. Pictures were taken at 0- and 48-h time points. Cell invasion assay was conducted as follows: the 6-well Transwell plates with a polycarbonate filter membrane of 8-mm pore size were used. Cells were seeded on the upper chamber in the serum-free medium, while medium containing 10% FBS was applied to the lower chamber as a chemoattractant. After 48 h incubating, cells were fixed with 4% polyoxymethylene and stained with 0.5% crystal violet. The invaded cells were counted by an inverted microscope (Olympus IX 71,Tokyo, Japan).

### Western blot analysis

Cells were collected and dissolved in radio-immunoprecipitation assay (RIPA) buffer and protein concentration was measured using the BCA protein assay kit (Beyotime). Protein in each sample was isolated by 15% SDS-PAGE and then imprinted on polyvinylidene fluoride (PVDF) membrane. The membranes were blocked and incubated with primary antibody at 4℃ overnight, followed by incubation with HRP (horseradish peroxidase)-conjugated secondary antibody at 37℃ for 2 h. The ECL (enhanced chemiluminescence) assay kit (Beyotime) was used for visualization.

### Nude mice tumorigenesis assay

Cells (5×10^7^) were mixed with Matrigel (1:1) and injected subcutaneously into BALB/c nude mice (male, about 4-week-old, Changzhou Cavens, Changzhou, China). The mice were randomly divided into four groups (n=4 per group): Vehicle, Erdafitinib (10 mg/kg), Stattic (5 mg/kg), Erdafitinib (10 mg/kg) + Stattic (5 mg/kg). When the tumor volumes reached approximately 100 mm^3^, the compounds were intraperitoneally injected once daily for 14 days. Body weight and tumor volumes were measured every other day. The animals' care and use were approved by the Laboratory Animal Ethics Committee of Jiangsu Institute of Nuclear Medicine (JSINM-2022-007).

### *In vivo* MicroPET imaging

For *in vivo* Micro-positron emission tomography (PET) imaging, mice received a tail vein injection of 100 μL of ^18^F-labelled fluorodeoxyglucose (^18^F-FDG, 3.7 MBq), and ten-minute static PET images were acquired at 1 h post-injection (n=4 per group). The PET images were quantitatively analyzed according to the previously reported methods 18.

### Histology and immunohistochemistry

After microPET imaging, the tumor tissues were acquired and used for H&E and immunohistochemistry (IHC) staining as previously reported 14. Imaging was analyzed using an epifluorescence microscope (Olympus, X81, Japan).

### Statistical analysis

All the experiments were performed independently at least three times, and the data are expressed as the mean ± SD. All statistical analysis were carried out using GraphPad Prism 6.0 Software. Statistical comparisons were conducted with the student's *t*-test between two groups and a one-way ANOVA followed by Tukey's post hoc test among three groups. A *p*-value of <0.05 was considered as statistically significant.

## Results

### STAT3 activation is remained upon Erdafitinib treatment in LUSC cells

**Figure 1:**
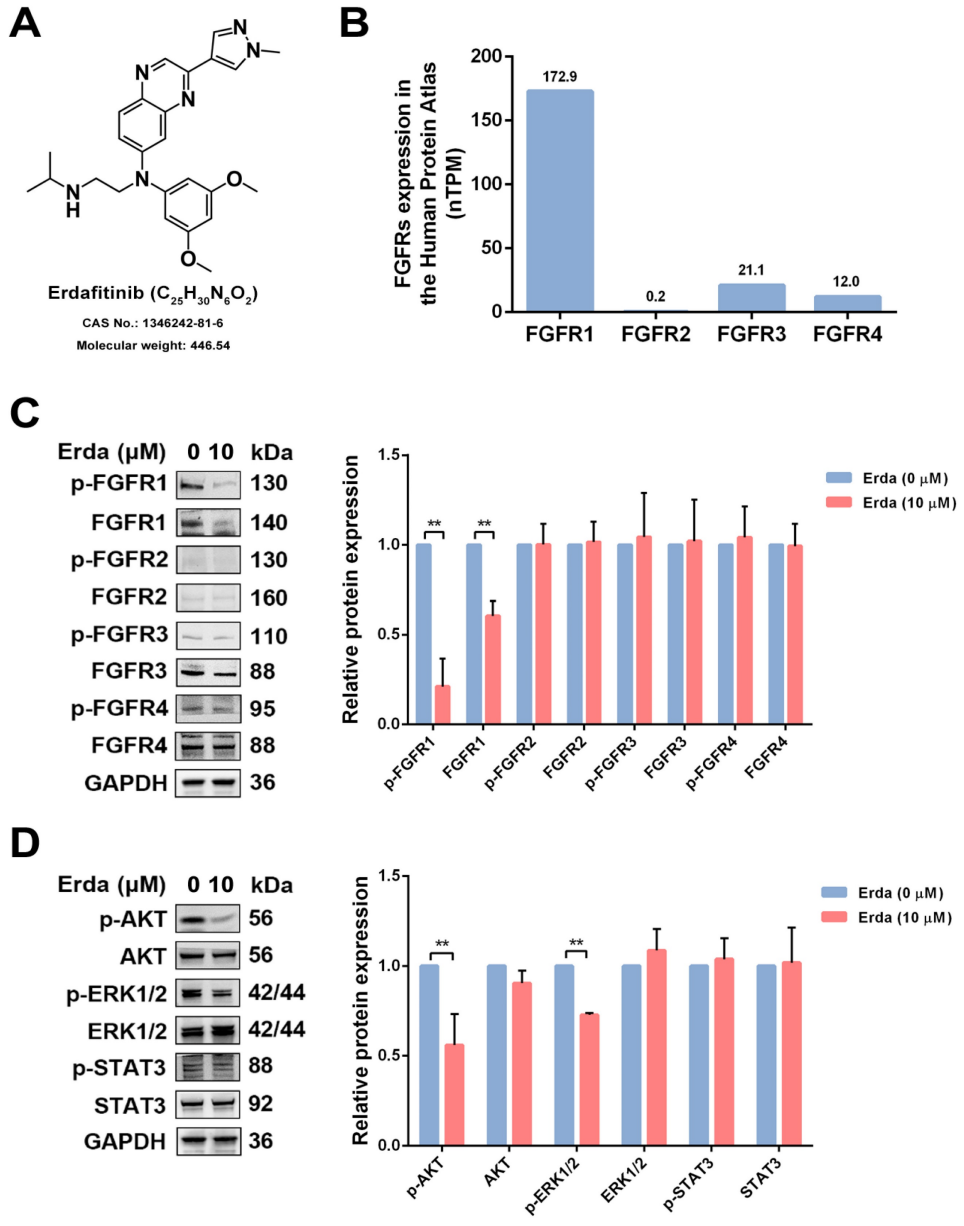
** The effects of Erdafitinib on FGFRs, AKT, MAPK, STAT3 signaling. A:** The chemical structure of Erdafitinib. **B:** The expressions of FGFRs in LUSC H520 cells from THE HUMANS PROTEIN ATLAS. **C:** H520 cells were treated with Erdafitinib (10 µM) for 24 h, and the expressions of FGFRs in H520 cells were assessed by Western blot analysis. **D:** H520 cells were treated with Erdafitinib (10 µM) for 24 h, and the expressions of molecules involved in AKT, MAPK, STAT3 signaling were assessed by Western blot analysis. Data was expressed as mean ± SD of three experiments and each experiment included triplicate repeats. ^**^p<0.01 *vs.* Erda (0 µM).

The chemical structure of Erdafitinib was shown in Figure 1A. To investigate the changes of FGFRs-related signaling in LUSC cells upon Erdafitinib treatment, the expressions of the FGFRs-related molecules were analyzed. As shown Figure 1B, the data from THE HUMANS PROTEIN ATLAS website showed that the protein expression of FGFR1 was the highest in H520 cells, compared to other FGFRs. LUSC H520 cells with Erdafitinib (10 µM) treatment for 24 h were selected for the further study due to the IC_50_ value of this drug (21.61 µM). As shown in Figure 1C, Erdafitinib treatment significantly down-regulated the expression of FGFR1 in H520 cells, indicating FGFR1 was the potential target of Erdafitinib. Then, the effects of Erdafitinib on AKT, MAPK, STAT3 signaling were further investigated. The results showed Erdafitinib significantly downregulated AKT and MAPK but had minimal impact on STAT3. It is plausible that the ineffective modulation on STAT3 may compromise the anti-cancer effect of Erdafitinib in LUSC H520 cells.

### The synergistic cytotoxic effects of Erdafitinib and Stattic on LUSC cells

**Figure 2:**
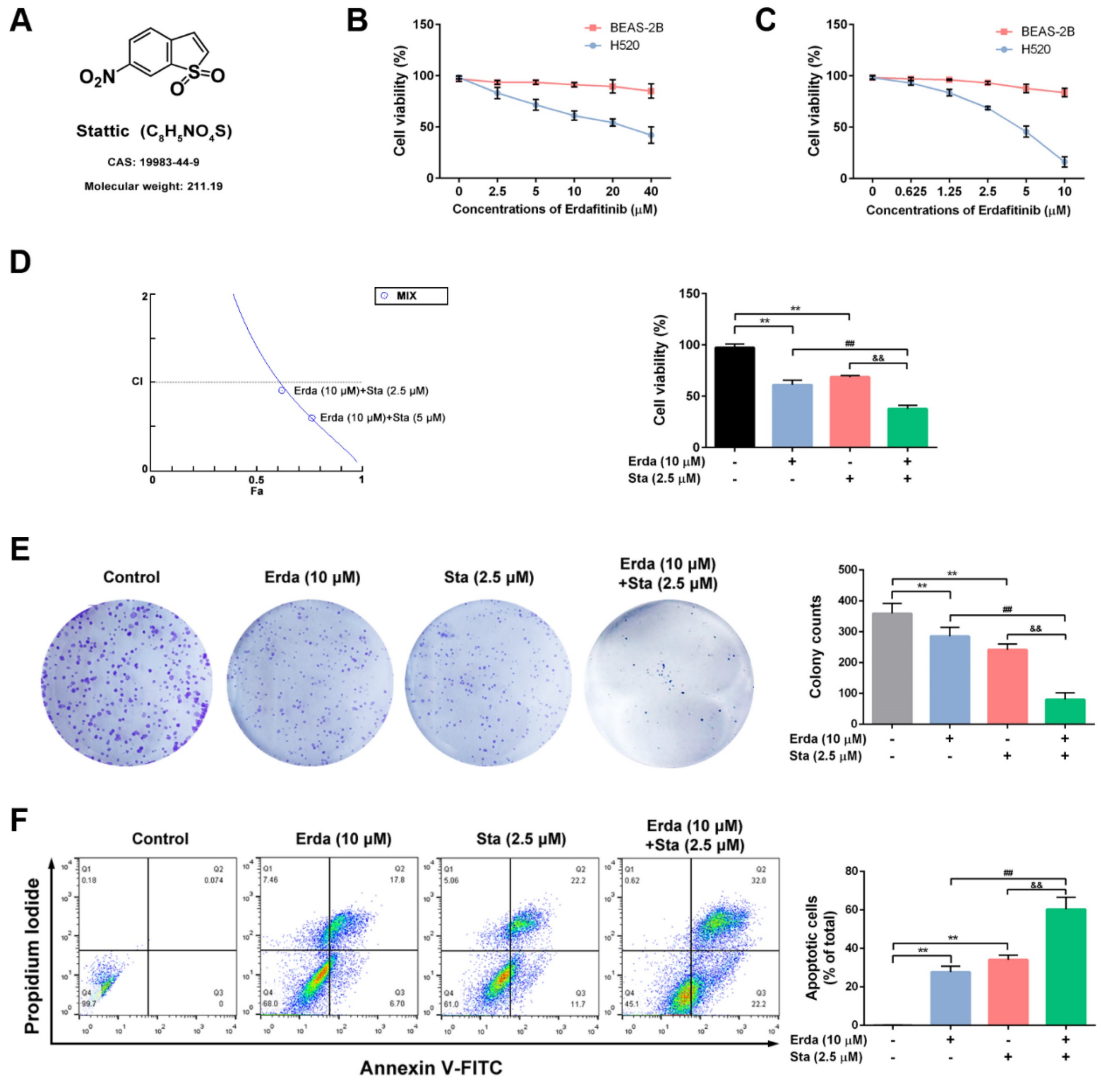
** The synergistic cytotoxic effects of Erdafitinib and Stattic on LUSC cells. A:** The chemical structure of Stattic. **B:** The effect of Erdafitinib on H520 and BEAS-2B cells was assessed by MTT assay. **C:** The effect of Stattic on H520 and BEAS-2B cells was assessed by MTT assay. **D:** The effects of Erdafitinib and Stattic on H520 cells were assessed by MTT assay, and the combination index (CI) was calculated. **E:** The concurrent effects of Erdafitinib and Stattic on H520 cells were assessed by colony formation assay. **F:** The concurrent pro-apoptosis effects of Erdafitinib and Stattic on H520 cells were assessed by flow cytometry analysis. The Annexin V+/PI- and Annexin V+/PI+ cells were considered as early and late apoptotic cells, respectively, and the sum of the above two was calculated as apoptotic cells. Data was expressed as mean ± SD of three experiments and each experiment included triplicate repeats. ^**^p<0.01 *vs.* Control, ^##^*p*<0.01 *vs.* Erda alone, ^&&^*p*<0.01 *vs.* Sta alone.

The chemical structure of Stattic (STAT3 inhibitor) was shown in Figure 2A. The synergistic cytotoxic effects of Erdafitinib and Stattic on LUSC cells were investigated using MTT assay, colony formation assay and flow cytometry analysis. As shown in Figure 2B&C, the IC_50_ of Erdafitinib (24 h treatment) was 21.61 μM for H520 cells, and the IC_50_ of Stattic (24 h treatment) was 3.97 μM for H520 cells, respectively. In addition, the IC_50_ of Erdafitinib and Stattic (24 h treatment) were more than 80 μM for human lung epithelial BEAS-2B cells. The CI value was estimated for Erdafitinib (10 µM) and Stattic (2.5 µM) co-treatment (24 h treatment) in H520 cells, which indicated a synergistic effect (CI=0.91, Figure 2D). As shown in Figure 2E, the data of colony formation assay is consistent with that of MTT assay. The synergistic pro-apoptotic effect of Erdafitinib (10 µM) and Stattic (2.5 µM) were also assessed using flow cytometry analysis. As shown in Figure 2F, Erdafitinib and Stattic co-treatment more significantly induced cell apoptosis compared to that of Erdafitinib or Stattic treatment alone group.

### The synergistic anti-metastatic effects of Erdafitinib and Stattic on LUSC cells

**Figure 3:**
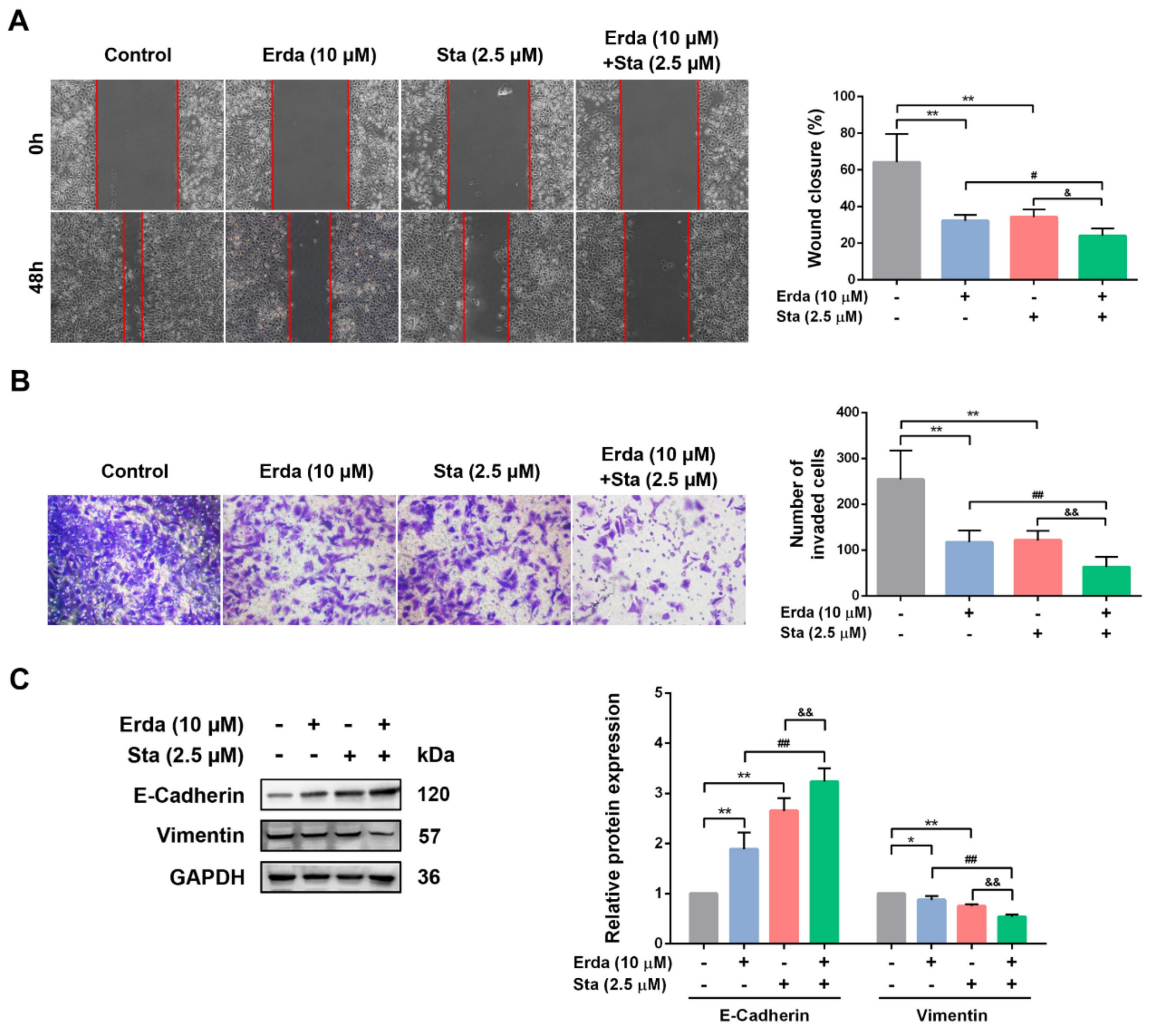
** The synergistic anti-metastatic effects of Erdafitinib and Stattic on H520 cells. A:** The concurrent anti-metastatic effects of Erdafitinib and Stattic on H520 cells was assessed by wound healing assay. **B:** The concurrent anti-invasive effects of Erdafitinib and Stattic on H520 cells was assessed by Transwell assay. **C:** The effects of Erdafitinib and Stattic co-treatment on the expressions of E-Cadherin and Vimentin in H520 cells. Data was expressed as mean ± SD of three experiments and each experiment included triplicate repeats. ^**^p<0.01 *vs.* Control, ^##^*p*<0.01 *vs.* Erda alone, ^&&^*p*<0.01 *vs.* Sta alone.

The synergistic anti-metastatic effect of Erdafitinib and Stattic (24 h treatment) in H520 cells was investigated using wound healing and transwell invasion assay. As shown in Figure 3A&B, Erdafitinib or Stattic treatment alone inhibited the migration and invasion of H520 cells; however, the co-treatment of Erdafitinib and Stattic exerted more pronounced anti-metastatic activity compared to either single treatment. E-cadherin and Vimentin are markers of epithelial mesenchymal transition (EMT), and which are involved in cancer cell metastasis. As shown in Figure 3C, Erdafitinib and Stattic co-treatment significantly up-regulated the expression of E-Cadherin, down-regulated the expression of Vimentin in H520 cells, compared to Erdafitinib or Stattic treatment alone (Figure 3C).

### Erdafitinib and Stattic exerts synergistic effects by inhibiting FGFR1/STAT3 activation in LUSC cells

**Figure 4:**
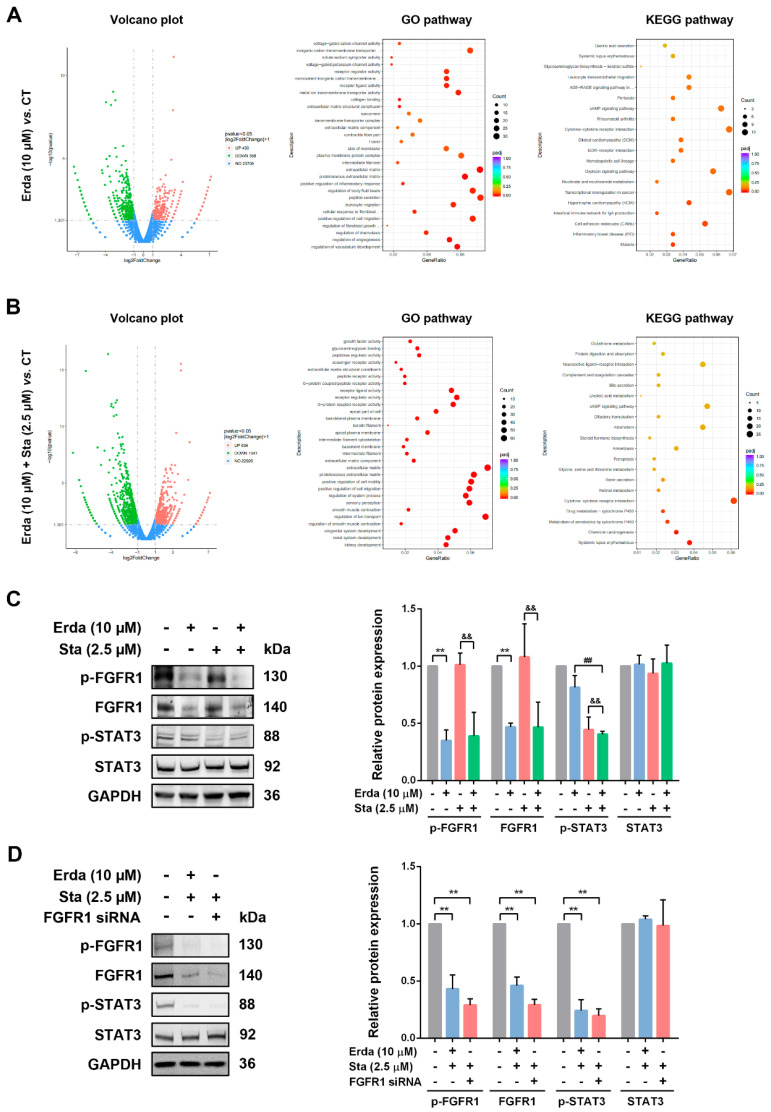
**The molecular mechanism responsible for the synergistic effects of Erdafitinib and Stattic on H520 cells. A:** Analysis of Volcano plots, GO pathway and KEGG pathway between the Erdafitinib (Erda,10 µM, 24 h) group and Control group. **B:** Analysis of Volcano plots, GO pathway and KEGG pathway between the Erdafitinib (Erda,10 µM, 24 h)+Stattic (Sta, 2.5 µM, 24 h) group and Control group. **C:** The concurrent effects of Erdafitinib (Erda,10 µM, 24 h) and Stattic (Sta, 2.5 µM, 24 h) on FGFR1/STAT3 signaling in H520 cells. **D:** The concurrent effects of FGFR1 knockdown and Stattic (Sta, 2.5 µM) on FGFR1/STAT3 signaling in H520 cells. Data was expressed as mean ± SD of three experiments and each experiment included triplicate repeats. ^**^p<0.01 *vs.* Control, ^##^*p*<0.01 *vs.* Erda alone, ^&&^*p*<0.01 *vs.* Sta alone.

The molecular mechanism responsible for the synergistic effects of Erdafitinib and Stattic was further investigated. As shown in Figure 4A, Volcano plot analysis revealed that there were 1018 differential expressed genes (DEGs) (430 genes up-regulation, and 588 genes down-regulation) were identified between Erdafitinib group and Control group, and there were 1355 differential expressed genes (DEGs) (519 genes up-regulation, and 836 genes down-regulation) were identified between Erdafitinib+Stattic group and Control group. As shown in Figure 4B&C, Erdafitinib+Stattic group showed the activation of IL-6/STAT3 signaling, indicating that Stattic exerts synergistic effect by affecting STAT3 activity in H520 cells. Then, validation experiments were conducted and the results showed that p-FGFR1/FGFR1 was down-regulated upon Erdafitinib treatment, while p-STAT3/STAT3 was down-regulated upon Stattic treatment in H520 cells. And p-FGFR1/FGFR1 and p-STAT3/STAT3 were both down-regulated in Erda+Sta group. In addition, cells with FGFR1 knockdown were treated with Stattic and the results were similar to that of Erdafitinib and Stattic co-treatment, indicating Erdafitinib and Stattic exerts synergistic effects by inhibiting FGFR1/STAT3 activation in H520 cells.

### The synergistic effects of Erdafitinib and Stattic against tumorigenesis* in vivo*

**Figure 5:**
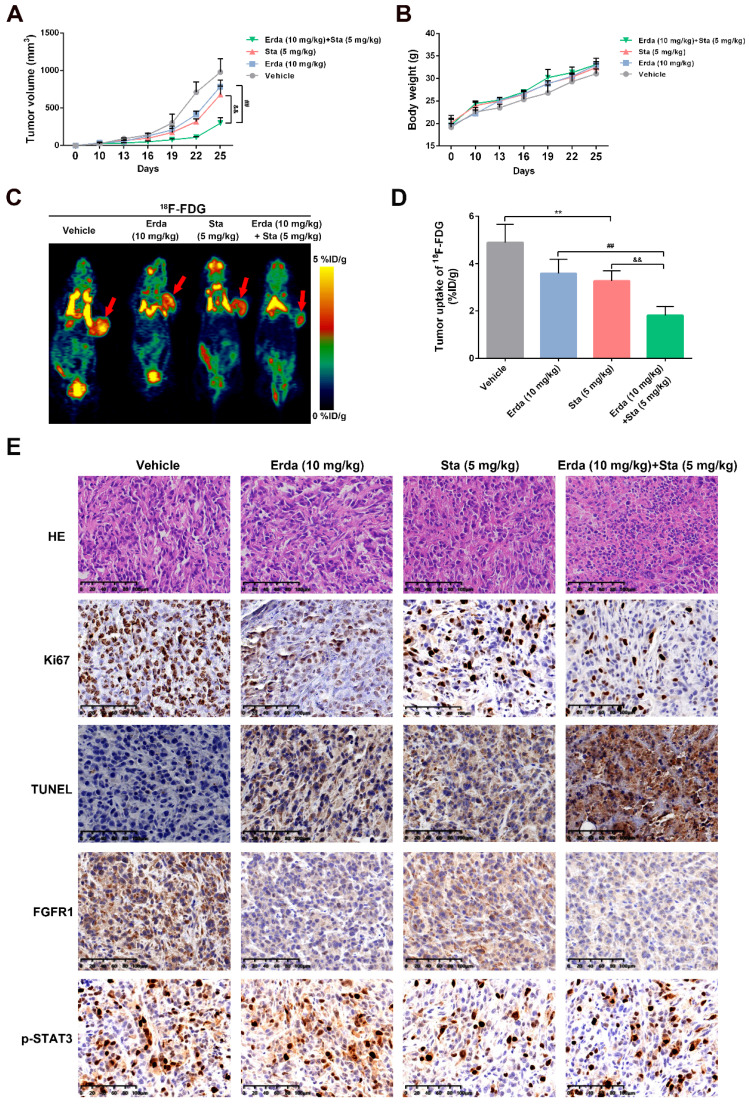
** The synergistic effects of Erdafitinib and Stattic against tumorigenesis* in vivo*.** H520 xenograft model was constructed and treated with Erdafitinib (Erda, 10 mg/kg/day), Stattic (Sta, 5 mg/kg/day), or their combination (10 mg/kg/day Erdafitinib, 5 mg/kg/day Stattic) for 2 weeks. **A:** Tumor volumes were measured every other day. **B:** Body weights were measured every other day. **C&D:** MicroPET was conducted using ^18^F-FDG. **E:** Representative images of hematoxylin-eosin (H&E), TUNEL, Ki67 and FGFR1/pSTAT3 staining in H520 xenografts. Data was expressed as mean ± SD of three experiments and each experiment included triplicate repeats. ^**^p<0.01 *vs.* Control,^ ##^*p*<0.01 *vs.* Erda alone, ^&&^*p*<0.01 *vs.* Sta alone.

To assess the synergistic effects of Erdafitinib and Stattic *in vivo*, a xenograft model using H520 cells was constructed. As shown in Figure 5A&B, Erdafitinib and Stattic showed the synergistic inhibitory effects on tumor growth and tumor volume. In addition, as shown in Figure 5C, the resected tumor tissues were stained with H&E, Ki67, TUNEL, FGFR1 and p-STAT3. H&E staining showed that Erdafitinib, Stattic or Erdafitinib and Stattic co-treatment induced morphological changes with the signs of cell necrosis and infiltration of inflammatory cells, decreased Ki67-positive cell and increased TUNEL-positive cells (indicative of cell apoptosis). And the combination of drugs shows a more pronounced effect. In addition, the data from IHC experiments showed that FGFR1 and p-STAT3 were both down-regulated upon co-treatment of Erdafitinib and Stattic, which were consistent to the *in vitro* results.

## Discussion

Targeted therapies in cancer treatment often inhibit specific molecular targets involved in tumor growth 19, 20. However, single-target therapies encounter many challenges in clinical settings due to rapidly emerged drug resistance likely through the activation of compensatory signaling pathways 21. Thus, multi-target therapies are more preferred with enhanced drug sensitivity and low risks of acquired drug resistance 22. In this study, we found that STAT3, one of downstream targets of FGFRs, was still activated upon Erdafitinib treatment in LUSC H520 cells, which might limit the anti-tumor activity of such drug. Then, Stattic, one STAT3 inhibitor, was found to have the synergistic effects with Erdafitinib by inhibiting STAT3 phosphorylation.

FGFRs, as members of receptor tyrosine kinases (RTKs), are transmembrane-type receptors with cytoplasmic tyrosine kinase domains, which transduce extracellular signals to a variety of intracellular signaling cascades, such as RAS-ERK, PI3K-AKT and JAK2/STAT3 5, 23. The FGFR inhibitors were widely developed for reducing phosphorylation of FGFRs and their downstream targets 24. Nintedanib was found to inhibit the proliferation of FGFR1-positive LUSC cell lines in association with attenuation of the FGFR1-ERK signaling pathway* in vitro* and *in vivo* 25. However, preliminary results from FGFR tyrosine kinase inhibitor trials in LUSC have shown that not all patients respond to therapy, how FGFR inhibitors can be combined with other targeted therapies or immunotherapies to improve patient outcome may help address this question. Erdafitinib has been shown to be effective in the treatment of cancers featured with amplifications, mutations and fusions of FGFRs 13. Our previous study has reported that Erdafitinib can effectively inhibit tumor growth of human lung adenocarcinoma cells; however, the effect of this drug in LUSC has never been explored. In the current study, Erdafitinib demonstrated a potent anti-cancer and anti-metastatic effect in *in vitro* and *in vivo* LUSC models. Upon assessing the protein expression of FGFRs downstream signaling factors in H520 cells, we found that STAT3 was resistant to Erdafitinib treatment. The IL-6/JAK/STAT3 pathway is aberrantly hyperactivated in many types of cancers and is generally associated with a poor clinical prognosis. The JAK/STAT3 signaling mediates the impact of IL-6 on tumor cell proliferation, survival, invasion, and metastasis as well as the suppression of anti-tumor immunity 26. Thus, STAT3 unresponsiveness might compromise the anti-tumor effect of Erdafitinib in LUSC. SH2 domain Stattic selectively inhibits activation, dimerization, and nuclear translocation of the SH2 domain of STAT3 and increases the apoptotic rate of STAT3-dependent lung cancer cell lines 27. In the current study, Stattic was applied together with Erdafitinib, which exhibited a synergistic effect via effectively inhibiting STAT3 in *in vitro* and *in vivo* LUSC models. Consistent results were obtained in cells with FGFR1 knockdown, indicating that simultaneous inhibition of FGFR1/STAT3 signaling greatly promotes the anti-cancer activity of Erdafitinib. In the future studies, it is desired that such combined therapeutic approach will be further verified with the combinations of Erdafitinib and other STAT3 inhibitors or STAT3 gene knockdown.

## Conclusion

Overall, the current study is the first attempt to evaluate the effectiveness of combined therapies of FGFR inhibitors (i.e. Erdafitinib) and other targeted therapies (i.e. Stattic) in LUSC, which regimen shows a promising and optimal effect in the treatment of FGFR-positive LUSC.
